# A Case of a Nonpetrous Cholesterol Granuloma Presenting as a Temporal Mass

**DOI:** 10.1155/2020/8872270

**Published:** 2020-12-14

**Authors:** Nathaniel Reeve, Jacob Kahane, Matthew Ng

**Affiliations:** ^1^University of Nevada Las Vegas, School of Medicine, Department of Otolaryngology—Head and Neck Surgery, Las Vegas, NO, USA; ^2^Louisiana State University, School of Medicine, Department of Otolaryngology—Head and Neck Surgery, New Orleans, LA, USA

## Abstract

**Objective:**

A case of a skull base cholesterol granuloma (CG) of the squamosal temporal bone. This is the first ever reported case of CG in a well-pneumatized squamous temporal bone.

**Design:**

Case report and review of the literature. *Discussion*. CG is a cystic mass typically found in the petrous apex and occasionally in the paranasal sinuses and orbit. Experience with the treatment of these expansile and inflammatory processes has largely been garnered from those occurring in the petrous apex, where they are surgically drained, via a transtympanic, transmastoid, or middle fossa approach. We report a case of cholesterol granuloma situated in the temporal fossa presenting as a temporal mass. The accessible location of this particular lesion made it amenable to total excision, avoiding the need for surgical drainage and possibility for recurrence.

**Conclusion:**

This case supports the theory of pathogenesis of such lesions typically occurring where pneumatized air spaces interface with bone marrow, in this case, where the reaches of pneumatized cells in the squamous portion of the temporal bone meet diploic bone.

## 1. Introduction

First described over 100 years ago, temporal bone CGs are defined pathologically by the presence of cholesterol crystals surrounded by multinucleated giant cells, round cell infiltration, and hemosiderin-laden macrophages [1]. These expansile lesions of the temporal bone are most often found at the petrous apex or tympanomastoid portions of the temporal bone. Pathogenesis of this entity has evolved over the last two decades. Jackler and Cho described two distinct theories of pathogenesis. The tympanomastoid CGs are likely due to impairment of ventilation of the middle ear and mastoid, causing hemorrhage and anaerobic metabolism of blood, resulting in hemosiderin deposits and other detritus in the middle ear. However, this classical obstruction-vacuum hypothesis does not explain the finding of CGs in the petrous apex. Rather, “exuberant” pneumatization of the petrous temporal bone allows for direct interaction of air cells with adjacent marrow space. The resulting hemorrhage into the pneumatized petrous apex allows for formation of the CG; this is known as the exposed marrow hypothesis [2].

The mainstay of surgical management for the petrous apex CG is achieving and maintaining drainage. While there exists some controversy as to whether the cyst wall should be removed, simple drainage is the current accepted intervention due to the difficulty to access region of the petrous apex and its proximity to vital skull base structures [3]. We describe the first described case of a CG occurring in a well pneumatized squamosal temporal bone and presenting as an expanding temporal mass.

## 2. Case Report

**Figure 1:**
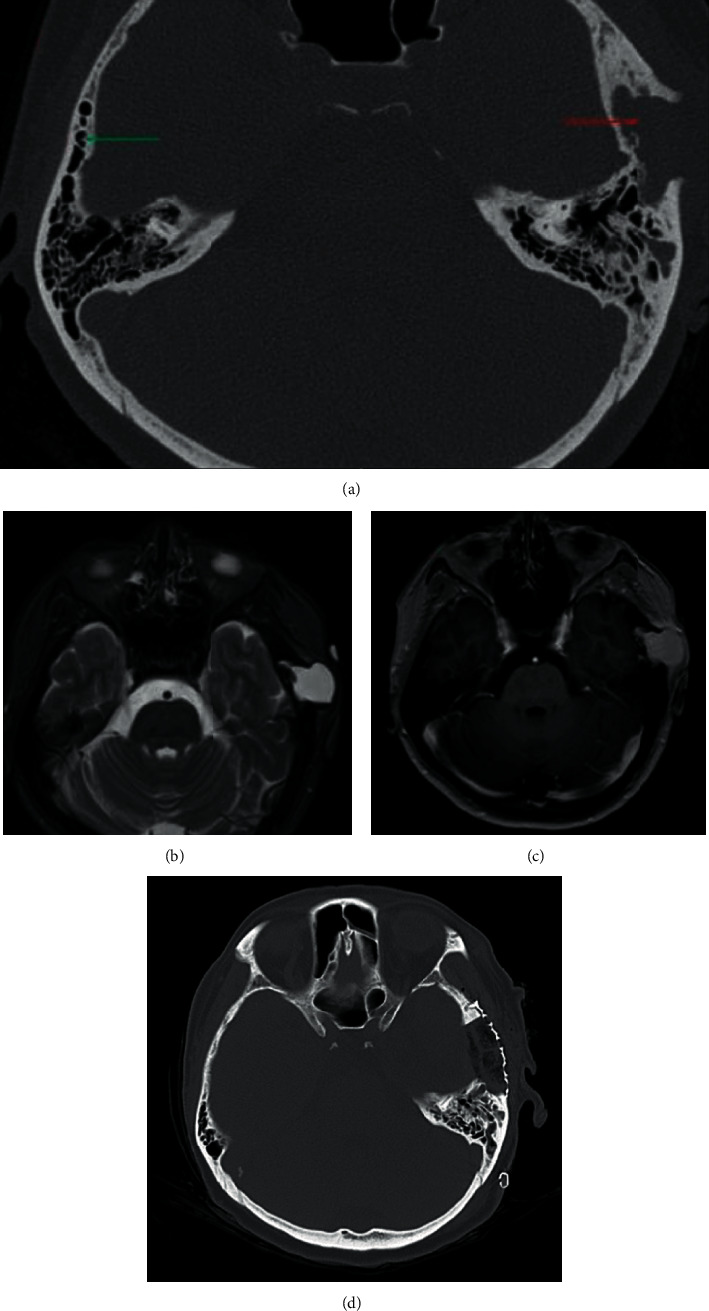
(a) Axial CT of the temporal bones showing a cystic mass of the left squamosal temporal bone disrupting the lateral cortex on the left (red arrow) and a well-pneumatized contralateral squamosal bone (green arrow). (b) T2-weighted axial MRI showing a fluid-filled mass at the left squamosal temporal bone. (c) T1 imaging reveals the lesion to be hyperintense and nonenhancing. (d) Postoperative CT showing complete resection with reconstruction via mesh cranioplasty.

A 67-year-old female presented due to a left temporal swelling of 5 months. She denied history of trauma, discomfort with chewing, audiologic concerns, or history of infection. Physical exam revealed a 2.5 cm fluctuant mass of the left temporal fossa. Subsequent computed tomography (CT) scan of the temporal bone showed a 2.3 cm cystic lesion of the left squamosal temporal bone with disruption of the lateral bony cortex (Figure 1(a)). MRI revealed a nonenhancing, T2 hyperintense lesion (Figures 1(b) and 1(c)). Ultrasound-guided fine-needle aspiration was nondiagnostic, revealing only cyst contents. The patient was taken to the operating room where a supra-auricular scalp flap was elevated. A temporoparietal craniectomy was performed to begin removal of the cystic mass en bloc. A limited superior mastoidectomy was performed to exenterate all soft tissues. The mastoidectomy defect was plugged using free muscle and bone wax prior to cranioplasty and closure. Pathologic review of the specimen showed a cyst with cholesterol crystals and hemosiderin-laden macrophages. After 6 months, there has been no recurrence or further complication.

## 3. Discussion

Cholesterol granulomas are expansile cystic lesions with the potential for osseous erosion that consists of cholesterol crystals within a fibrous capsule [1]. While CGs have been reported in multiple pneumatized bone tracts of the head and neck (including the orbit and ethmoid air cells), the majority occur in the petrous apex or tympanomastoid cavity of the temporal bone [1, 4, 5]. Their incidence in the squamosal temporal bone has yet to be reported, possibly due to the relatively low volume of aeration typically found in this area. Diagnosis of CG is often precipitated by symptoms of cranial nerve involvement including headache, hearing loss, dizziness, facial numbness, and diplopia [2, 3]. MRI characteristics are unique and include hyperintense signal on T1- and T2-weighted imaging with absence of enhancement. There are two mechanisms for CG formation currently supported by the literature. The first postulates that restricted airflow in pneumatized temporal bone cells results in negative pressure, followed by inflammation, angiogenesis, and blood vessel breach with hemoglobin deposition. Subsequent dissolution of hemoglobin byproducts results in continual inflammation and aggregation of cholesterol crystals. However, a squamosal temporal bone CG, in the setting of a well-pneumatized and unobstructed mastoid, supports the second proposed mechanism. This is the exposed marrow hypothesis, which states that exposed marrow allows for coaptation of the marrow and mucosal spaces. This is then followed by hemorrhage and a foreign body reaction to the hemorrhagic breakdown products. In light of this evidence, the diagnosis of CG should be considered in any situation where a temporal bone lesion is present in an area capable of pneumatization.

CGs are treated with either surgical drainage or marsupialization, as access to these lesions can be difficult. Many occur in the petrous apex and necessitate complex surgical approaches, and a wait and scan approach has become accepted in many instances when tolerable [6]. Complications of surgical drainage include sensorineural hearing loss, facial nerve injury, CSF leak, and carotid artery injury [6]. Recurrence rates requiring reoperation range from 9% to above 18% [6]. However, the presence of a CG in an aerated squamosal temporal bone allows for the unique opportunity for complete removal with relatively little morbidity. Squamosal temporal bone CG has not been reported previously, and the existence of this clinicopathologic entity lends credence to the exposed marrow hypothesis for CG formation over the initially posited obstructed air cell hypothesis. As a result, CG should be a differential consideration in the setting of any temporal bone lesion occurring in a well-pneumatized region. Squamosal temporal bone CGs are particularly amenable to complete removal, rather than drainage, which should prevent recurrence.
